# An advanced expiratory circuit for the recovery of perfluorocarbon liquid from non-saturated perfluorocarbon vapour during partial liquid ventilation: an experimental model

**DOI:** 10.1186/1475-925X-5-7

**Published:** 2006-02-03

**Authors:** Kimble R Dunster, Mark W Davies, John F Fraser

**Affiliations:** 1 Grantley Stable Neonatal Unit, Royal Women's Hospital, Brisbane, Queensland, Australia; 2 Medical Engineering, Queensland University of Technology, Brisbane, Queensland, Australia; 3 Department of Paediatrics and Child Health, The University of Queensland, Brisbane, Queensland, Australia; 4 Department of Intensive Care Medicine, The Prince Charles Hospital, Brisbane, Queensland, Australia

## Abstract

**Background:**

The loss of perfluorocarbon (PFC) vapour in the expired gases during partial liquid ventilation should be minimized both to prevent perfluorocarbon vapour entering the atmosphere and to re-use the recovered PFC liquid.

Using a substantially modified design of our previously described condenser, we aimed to determine how much perfluorocarbon liquid could be recovered from gases containing PFC and water vapour, at concentrations found during partial liquid ventilation, and to determine if the amount recovered differed with background flow rate (at flow rates suitable for use in neonates).

**Methods:**

The expiratory line of a standard ventilator circuit set-up was mimicked, with the addition of two condensers. Perfluorocarbon (30 mL of FC-77) and water vapour, at concentrations found during partial liquid ventilation, were passed through the circuit at a number of flow rates and the percentage recovery of the liquids measured.

**Results:**

From 14.2 mL (47%) to 27.3 mL (91%) of the infused 30 mL of FC-77 was recovered at the flow rates studied. Significantly higher FC-77 recovery was obtained at lower flow rates (ANOVA with Bonferroni's multiple comparison test, p < 0.0001). As a percentage of the theoretical maximum recovery, 64 to 95% of the FC-77 was recovered. Statistically significantly less FC-77 was recovered at 5 Lmin^-1 ^(ANOVA with Bonferroni's multiple comparison test, p < 0.0001). Amounts of perfluorocarbon vapour recovered were 47%, 50%, 81% and 91% at flow rates of 10, 5, 2 and 1 Lmin^-1^, respectively.

**Conclusion:**

Using two condensers in series 47% to 91% of perfluorocarbon liquid can be recovered, from gases containing perfluorocarbon and water vapour, at concentrations found during partial liquid ventilation.

## Background

Partial liquid ventilation involves filling the lungs to functional residual capacity with a liquid, in which oxygen and carbon dioxide are soluble, while continuing ventilation with a conventional gas ventilator.

Perfluorocarbon (PFC) liquids seem to be physiologically ideal for liquid ventilation, yet they have two major drawbacks. The first is environmental and the second major concern is cost. All PFCs are photochemically stable, have a high global warming potential [1] and are contributors to the greenhouse effect [2]. It would seem prudent, therefore, to limit the losses of PFC during partial liquid ventilation, both to prevent PFC vapour entering the atmosphere and to re-use the recovered PFC liquid. As all the lost PFC is in the exhaled gases, an opportunity exists to recover the PFC.

We have previously shown that up to 74% of PFC can be recovered from saturated vapour using a simple chilled condenser [3]. However, a major problem with the experimental set-up and condenser for that study was that it condensed the PFC from gas that was fully saturated with PFC vapour and not humidified; a situation that would not be found during the clinical use of partial liquid ventilation. During partial liquid ventilation the gas in the expiration limb consists of a mixture of expired gases (which are saturated with PFC vapour) and the bias flow. As such, the gas in the expiration limb would not be saturated with PFC vapour and during partial liquid ventilation the amount of PFC recoverable with a simple chilled condenser would be limited. The presence of water vapour would also complicate the process because, at temperatures at which significant PFC vapour condenses, water freezes.

Using a substantially modified design of our previously described condenser, we aimed to see how much PFC liquid could be recovered from gases containing PFC and water vapour, at concentrations found clinically during partial liquid ventilation, and to determine if the amount recovered differed with background flow rate (at flow rates suitable for use in neonates).

## Methods

**Figure 1:**
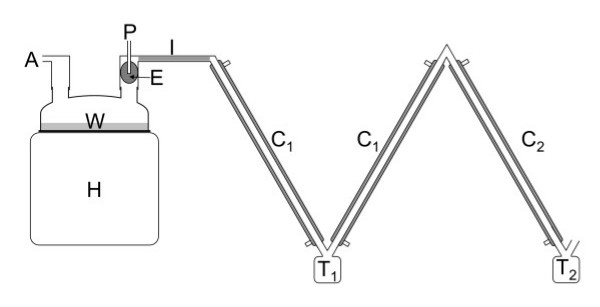
**Experimental apparatus**. A = air inlet, H = humidifier, W = water, P = perfluorocarbon inlet, E = perfluorocarbon evaporator, I = heated inspiratory line, C_1 _= 1°C condenser, C_2 _= -30°C condenser, T_1_, T_2 _= traps.

The experimental apparatus is shown in Figure 1. The purpose of the apparatus was to mimic the expiratory line of a standard ventilator circuit set-up and provide a gas mixture that mimicked the water and FC-77 concentrations found in the expired gases during partial liquid ventilation of an approximately 3.5 kg infant [4]. This was achieved using a bias gas flow and evaporating FC-77 into the flow at the replacement rate used during partial liquid ventilation of an infant.

To produce the required gas mixture a humidifier (MR600, Fisher & Paykel Healthcare, Auckland, New Zealand) was set to give a chamber outlet temperature of 39°C and an inspiratory line outlet temperature of 37°C, as is commonly used in clinical practice. A self-filling chamber (MR290, Fisher & Paykel Healthcare, Auckland, New Zealand) and conventional heated inspiratory circuit (Fisher & Paykel Healthcare, Auckland, New Zealand) were used. A sponge (#00–005, Multigate Medical Products, Sydney, Australia) was placed in the outlet of the humidifier chamber as an FC-77 evaporator. Air was delivered to the chamber at flows of 1 Lmin^-1^, 2 Lmin^-1^, 5 Lmin^-1 ^and 10 Lmin^-1^. For each test 30 mL of FC-77 (vapour pressure = 9.99 kPa at 37°C) [5,6] was infused, using a syringe pump, onto the evaporator over a one hour period. Measurements were performed in triplicate for each flow rate.

The air, water vapour and FC-77 vapour from the chamber passed through two series-connected condensers. The first condenser was designed to remove water vapour from the gas mixture, in order to prevent the formation of ice in the second condenser. The first condenser consisted of two coaxial tubes (modified from prototype ventilator circuits, 10 mm internal diameter, smooth bore, 1 m long, Fisher & Paykel Healthcare, Auckland, New Zealand). The outer coaxial tube was cooled to 1°C (2219 Thermostatic Circulator, LKB, Bromma, Sweden) and the gas mixture passed through the inner tube with a total condensing surface of 0.063 m^2^.

The second condenser was designed to remove PFC from the gas mixture. It consisted of a 1 m long stainless steel tube (13 mm internal diameter, 1 mm wall thickness) with an outer jacket of 20 mm PVC tubing cooled to -30°C (9102 Circulator, Polyscience, Niles, Illinois, USA) and the gas mixture passed through the inner tube with a condensing surface of 0.041 m^2^.

At the conclusion of each experiment the condensers were warmed to ~2°C to ensure any water ice had melted, the volumes of water and FC-77 in each trap were measured and the total volumes calculated. The percentage of theoretical maximum recovery was calculated (see below). The humidifier chamber was visually inspected for the presence of any residual FC-77. The system was flushed with dry air for at least 60 minutes between experiments.

At all flow rates studied the gas stream would be saturated with water vapour which has a density of 44 mgL^-1 ^at 37°C and 5.2 mgL^-1 ^at 1°C [7]. Therefore a theoretical maximum of 38.8 mg of water could be condensed for each litre of air passing through the condensers. This corresponds to a maximum water recovery of 2.3 mL, 4.7 mL, 11.6 mL and 23.3 mL at 1 Lmin^-1^, 2 Lmin^-1^, 5 Lmin^-1 ^and 10 Lmin^-1 ^respectively.

The saturated vapour density of FC-77 is 1.6 gL^-1 ^at 37°C, 0.28 gL^-1 ^at 1°C and 0.04 gL^-1 ^at -30°C [5]. In these experiments the vapour was not saturated and the pre-condenser vapour density was calculated by dividing the weight of FC-77 infused (53.4 g) by the total volume of air passed during the experiment. Thus the FC-77 vapour densities were 0.89 gL^-1^, 0.45 gL^-1^, 0.18 gL^-1 ^and 0.09 gL^-1 ^at 1 Lmin^-1^, 2 Lmin^-1^, 5 Lmin^-1 ^and 10 Lmin^-1 ^respectively. The condensers cannot reduce the vapour density below the saturation vapour density at the condenser temperature, dictating the maximum amount of FC-77 that can theoretically be condensed. The maximum possible FC-77 recovery was 28.7 mL, 27.3 mL, 23.3 mL and 16.5 mL at 1 Lmin^-1^, 2 Lmin^-1^, 5 Lmin^-1 ^and 10 Lmin^-1 ^respectively. Statistical comparisons were made using ANOVA with Bonferroni's multiple comparison test.

## Results

**Figure 2:**
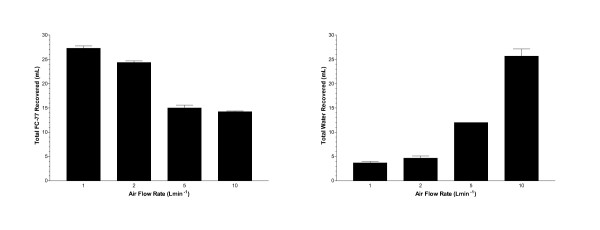
**Recovered Volumes**. Volumes of FC-77 and water recovered at different flow rates (mean ± sem). For FC-77, all pairs except 5 and 10 Lmin^-1 ^are significantly different (p < 0.05). For water, all pairs except 1 and 2 Lmin^-1 ^are significantly different (p < 0.05).

No unevaporated FC-77 remained in the humidifier chamber at any flow rate. From 14.2 mL (47%) to 27.3 mL (91%) of the infused 30 mL of FC-77 was recovered at the flow rates studied, and from 4 to 26 mL of water was recovered (Figure 2). Significantly higher FC-77 recovery was obtained at lower flow rates (p < 0.0001). Significantly higher water recovery was obtained at higher flow rates (p < 0.0001).

**Figure 3:**
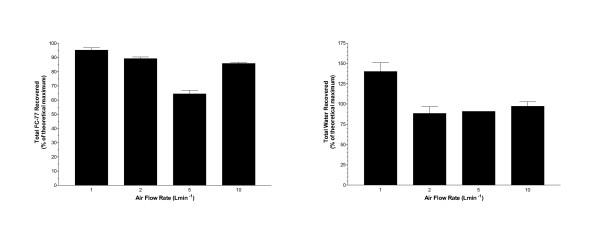
**Recovery Percentages**. Percentages of the theoretical maximum recovery for FC-77 and water at different flow rates (mean ± sem). For FC-77, 1 and 2; 2 and 10 Lmin^-1 ^are significantly different (p < 0.05). For water, 1 Lmin^-1 ^is significantly different (p < 0.05) from all other rates.

As a percentage of the theoretical maximum recovery, mean recoveries were 64 to 95% for FC-77 and 88 to 140% for water (Figure 3). Statistically significantly less FC-77 was recovered at 5 Lmin^-1 ^(p < 0.0001) and significantly higher water recovery was obtained at 1 Lmin^-1 ^(p < 0.01).

## Discussion

For economic and environmental reasons it is desirable to minimise the loss of PFCs during partial and total liquid ventilation. Some of the properties of PFCs that make them ideal for liquid ventilation also make recovery and re-use possible. FC-77 has a density of 1.78 gcm^-3 ^and is not miscible with water [5] and thus they can be easily separated from each other. Being hydrophobic PFCs do not support bacterial growth. No chemical decomposition occurs at body temperature and biological materials will not dissolve in PFCs. As such, the PFCs can easily be washed and filtered for reuse in the same patient. All PFC loss occurs through evaporation and the PFC vapour all passes through the expiratory line of the ventilator circuit. This provides an opportunity to condense the PFC vapour from expired gases.

Perfluorooctyl bromide [8,9] loss rates of 2 to 6 ml/kg/h and FC-77 [4] loss rates of around 7 ml/kg/hr have been reported for partial liquid ventilation.

The amount of vapour passing into the exhalation line during partial liquid ventilation depends primarily on the surface area of the PFC-air interface and the temperature. Hence, the concentration in the expiratory line depends on the amount of PFC and the gas flow rate. The vapour pressure (perfluorooctyl bromide (1.47 kPa at 37°C) [5] and FC-77 (9.99 kPa at 37°C) [5,6]) and, hence, the vapour density, of the PFC will impact on the condensation efficiency. Having a lower vapour pressure, perfluorooctyl bromide evaporates less than FC-77, but also will more readily condense. The amount of perfluorooctyl bromide recoverable will need to be tested in future experiments.

Using a humidified circuit, we studied the recovery of FC-77 with a loss rate of 30 ml/hr, corresponding to the loss rate expected for an approximately 3.5 kg infant [4]. A previous study showed recoveries of up to 74% when examining the recovery of FC-77 from saturated, non humidified, vapour – a situation which would not be found in clinical partial or total liquid ventilation [3].

Two series-connected condensers were employed, the first at 1°C to remove water (which would solidify in the second condenser) and the second at -30°C to condense the FC-77.

Water is readily condensed at 1°C and over 88% of the theoretical maximum [7], was recovered at all flow rates studied. The greater than theoretical recovery at 1 Lmin^-1 ^may be attributed to the carry over of a few drops of particulate water, the humidifier over heating slightly at this low flow rate, or inaccuracies in the flowmeter.

A small amount of water (up to 3 mL) was found in the second (-30°C) condenser, particularly at higher flow rates. This water forms ice in the second condenser and may block the condenser. In research or clinical practice it would be necessary to have two condensers which could be used alternatively to allow any water ice to melt. Condenser switching would be automated based on an increase in differential pressure across the condenser. Additional safety 'blow-off' valves would also used to prevent excessive circuit pressures in the event of condenser blockage.

In absolute terms, 47% to 91% of the evaporated PFC was recovered. Over 65% of the theoretical maximum amount of PFC was recovered at all flow rates studied. At the commonly used flow rate of 10 Lmin^-1^, 86% of the theoretical maximum was recovered. The drop in recovery at 5 and 10 Lmin^-1 ^can be attributed to either reduced efficiency of the second condenser at these high flow rates or a probable change from laminar to turbulent flow. Various strategies could be employed to improve the efficiency of the second condenser at the higher flow rates: increasing the length of the condenser would increase the surface area; packing the condenser with stainless steel wool would cause turbulence in the flow, as well as increasing the surface area for condensation.

Further cooling of the second condenser would also increase the recovery. Cooling to dry ice temperature (-78°C) would increase the theoretical maximum recovery of FC-77 to >99% of the infused volume at a flow rate of 10 Lmin^-1^, compared with a theoretical maximum recovery of 55% of the infused volume with a condenser temperature of -30°C [5]. -30°C was employed in the current study as the lowest temperature obtainable with the apparatus.

Using a complicated rebreathing system, Schhrader et al obtained a 90% recovery rate for perfluorooctyl bromide in animal studies utilizing partial liquid ventilation [10]. Specially designed heat and "moisture" exchangers fitted to the endotracheal tube may be also used to minimize PFC loss during partial liquid ventilation, with 60% more perfluorooctyl bromide retained than without the exchanger [11]. These methods, however, all require complicated ventilator circuits and equipment or add dead space that would be unacceptable in neonatal ventilation.

This study represents a substantial advancement in the development of a perfluorocarbon vapour recovery circuit. We have been able to overcome the problems of expired gases not fully saturated with PFC vapour and yet fully saturated with water vapour. We have demonstrated this at flow rates that are used in neonatal ventilation. We have further refined the design of the expiratory circuit with a small surface area, which would not significantly alter conventional ventilator function, that achieves PFC recovery similar to other methods. For the first time, recovery of PFC from expired gases that are not saturated with PFC, and saturated with water, have been demonstrated. Further studies will be necessary to determine the PFC recovery that can be achieved with flow rates and ventilator circuits suitable for paediatric and adult ventilation. Whilst recovery would be less at higher flow rates in bigger patients some recovery would still be possible, and some is better than none. Also the increase in size of the patients would make modifications possible to improve condensation.

## Conclusion

Using two series connected condensers 47% to 91%, of PFC liquid (FC-77) can be recovered from clinical relevant gas mixtures without altering the function of the expiratory line of a ventilator circuit. Further development of the system could allow re-use of the PFC recovered during partial liquid ventilation; thus curtailing loss of PFC to atmosphere and the potential high costs of partial liquid ventilation.

## List of abbreviations

PFC – perfluorocarbon

ANOVA – analysis of variance

## Competing interests

The author(s) declare that they have no competing interests.

## Authors' contributions

KRD conceived the study, constructed the apparatus, carried out the experiments, performed the statistical analysis and drafted the manuscript. MWD conceived the study, participated in its design and coordination, assisted in the statistical analysis and revised the manuscript. JFF assisted in data analysis and interpretation, and revised the manuscript. All authors read and approved the final manuscript.
